# Self-Standing Doughs of SiAlONs Enable Low-Volume Production through Green Machining

**DOI:** 10.1021/acsomega.6c05186

**Published:** 2026-07-23

**Authors:** Ayse Ay, Gokhan Kula, Burcin Gul, Ceylin Isiklar, Eminenur Saritas, Alpagut Kara, Ozge Akbulut

**Affiliations:** † Faculty of Engineering and Natural Sciences, 52991 Sabanci University, Istanbul 34956, Turkiye; ‡ 395780 MDA Advanced Ceramics Inc., 75.Yil OSB Mah., Anadolu Cad. No: 2, Odunpazari, Eskisehir 26250, Turkiye; § Department of Materials Science and Engineering, Eskisehir Technical University, Eskisehir 26555, Turkiye

## Abstract

SiAlONs are silicon-, aluminum-, oxygen-, and nitrogen-based ceramic alloys used in high-performance applications for their thermal and mechanical properties. Their use requires custom geometries, and near-net shaping is typically carried out at the green state. This study reports the preparation of self-standing SiAlON doughs with a 0.9Y-0.5Sm-0.5Ca composition, produced by hand-mixing in under 2 min. These clay-like doughs overcome the limitations of powder-pressing and slurry-based methods. After hand-shaping or molding, they form green bodies suitable for CNC machining for low-volume production and custom-based design. A single additive, a poly­(ethylene glycol)-grafted random copolymer of acrylic acid and 2-acrylamido-2-methylpropanesulfonic acid, was used at 2 wt % to impart malleability to 72.7 wt % SiAlON suspensions. These doughs exhibited a flexural strength of ∼0.5 MPa, enabling machining without tool wear while maintaining integrity. Machining was performed at room temperature after drying. Mechanical and microstructural properties were compared with those of a wear part made by uniaxial pressing. After gas-pressure sintering, hardness and Vickers toughness values of dough-derived samples (13.48 ± 0.35 GPa, 6.22 ± 0.28 MPa·m^1/2^) were comparable to the uniaxially pressed samples (14.40 ± 0.39 GPa, 6.40 ± 0.40 MPa·m^1/2^). The doughs yield ceramics with 19% isotropic shrinkage and 3.24 g·cm^–3^ density (∼99.99% theoretical) postsintering, enabling efficient fabrication and prototyping of complex shapes.

## Graphical abstract

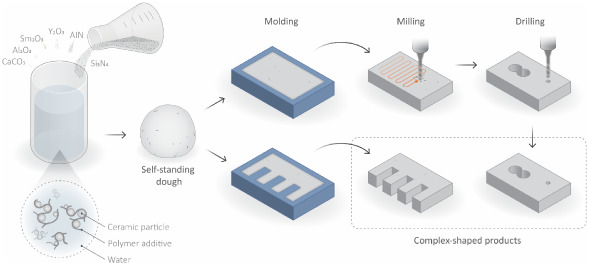

## 1 Introduction

Characterized by exceptional abrasion resistance, thermal stability, and mechanical strength, silicon nitride (Si_3_N_4_) is widely utilized in challenging applications such as engine components, cutting tools, and wear-resistant parts.^1–6^ To enhance the mechanical and thermal properties of silicon nitride, aluminum, oxygen, and certain metal oxides are added to its formulation.^6–9^ These formulations, termed SiAlONs, achieve higher densification and provide improved thermal shock and wear resistance.^10–13^

SiAlONs are often employed to fabricate pins, nozzles, cutting tools, extrusion dies, and weld rolls in custom sizes and low volumes, driven by the distinct demands of various industries.^14–17^ Conventional mass-production techniques, including uniaxial pressing, cold isostatic pressing, and slip casting are restricted to basic geometries and are inadequate for fabricating components with complex features such as holes, threads, or grooves. Therefore, to reach the final form, semifinished SiAlON products are near-net shaped at the green or sintered state through machining; yet, postsintering machining is constrained by the brittle behavior of ceramics, which leads to significant material loss and tool wear.^18–23^ On the other hand, green machining provides a lower-energy route owing to the softness of the powder compact at the green state.^24–27^ In current practice, ceramic green bodies are typically formed by compressing powders into predefined shapes, a process that requires the incorporation of binders, often in relatively large amounts (3–40 vol %),^28–31^ to attain enough strength for enduring machining forces.

We have recently demonstrated imparting a dough-like rheology to the highly loaded suspensions of advanced ceramics (more than 60 wt %) through the incorporation of a single additive (less than 2 wt %), a random copolymer. In this methodology, the additive is composed of acrylic acid and a comonomer that is chosen to modify the ionicity of the polymer backbone. Sometimes poly­(ethylene glycol) is included in the system as a graft, exploiting steric hindrance to further tune the packing behavior of the suspension. We rely on polymer bridging to enable uniform coagulation, without phase separation, and generate a self-standing, malleable dough that can then be pushed into a mold or shaped by hand (Supporting Information, Video S1 and Figure 1).

**Figure 1:**
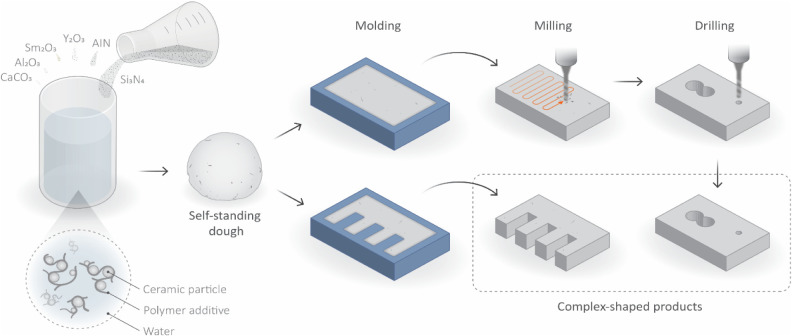
Process flow of the fabrication of complex-shaped objects from malleable, self-standing doughs comprising a multicomponent ceramic system.

Until now, we have prepared the doughs of single oxides such as alumina,^34^ zirconia,^32^ and magnesia.^33^ Here, for the first time in the literature, a multicomponent system, a SiAlON dough, is formed through polymer bridging-induced uniform coagulation. This homogeneous coagulation prevents phase separation and rapidly transforms a suspension of multiple particles into a self-standing, malleable green body. We employed a specific α–β SiAlON composition that was previously optimized to achieve full densification, where (i) CaCO_3_ is utilized in the Y–Sm–Ca multication system to obtain α-SiAlON, (ii) Y_2_O_3_ is added as a sintering aid, and (iii) Sm_2_O_3_ is incorporated to promote elongated β-SiAlON grains, thereby improving the fracture toughness of Si_3_N_4_.^35,36^ In this study, we used a poly­(ethylene glycol) (PEG)-grafted copolymer of 2-acrylamido-2-methylpropanesulfonic acid (AMPS) and acrylic acid (AA). To determine the minimum copolymer amount to consolidate highly loaded suspensions, we have carried out electrokinetic and rheological characterization. A ∼72.7 wt % aqueous SiAlON suspension in the presence of a 2 wt % additive with respect to the solid loading generated the best dough in terms of ease of processing and sustaining a crack-free surface (Figure 1).

Following gas pressure sintering, the samples showed an isotropic linear shrinkage of ∼19% from the dried state and reached a sintered density of ∼3.24 g·cm^–3^, which corresponds to ∼99.99% of theoretical density. The postsintering microstructure of polished surfaces was assessed via scanning electron microscopy (SEM). X-ray diffraction spectroscopy was used to estimate the percentage of the α- and β-phases of SiAlON. The Vickers indentation hardness of the sintered sample was compared with a wear part that was produced through pressing of the same powder and no significant difference was observed.

We believe shifting from the current industrial standard (e.g., compacted powder/binder mix) to malleable doughs could be the key for building resilient low-number manufacturing and prototyping schemes for advanced ceramics. These doughs do not require expensive molds or setups yet can easily comply with traditional machining. In contrast to conventional powder-compaction routes, the proposed approach eliminates the need for rigid green bodies and dedicated tooling, making it particularly attractive for low-volume manufacturing and rapid prototyping. The SiAlON dough has a much lower flexural strength of ∼0.5 MPa compared to the other reported powder/binder mixes of SiAlONs (20–90 MPa)^37–39^ allowing machining with lower forces while keeping its integrity due to its coagulated nature. The combination of direct shapeability, green-state machinability, and near-theoretical densification distinguishes the proposed approach from previously reported SiAlON processing routes. Overall, the expansion to nitrides in the materials portfolio of self-standing doughs is promising for developing energy-, cost-, and resource-efficient routes for the processing of advanced ceramics.

## 2 Experimental Section

### 2.1 Materials

The raw materials used were as follows: High-purity α-Si_3_N_4_ powder containing 1.3 wt % O (P95H, Vesta Corp.), Y_2_O_3_ (>99.9%, Reetec), AlN powder containing 1.6 wt % O (H grade, Tokuyama Corp.), α-Al_2_O_3_ powder (P172LSB grade, Alteo), CaCO_3_ (>99.75%, Reidel-de Haën), and Sm_2_O_3_ (>99.9%, Stanford Materials Corp.). AMPS (99%), AA (99%), and potassium peroxydisulfate (KPS, ≥99.0%) were purchased from Sigma-Aldrich. PEG (Mw = 1000 g·mol^–1^), hydrochloric acid (HCl, 37%), sodium hydroxide (NaOH, 97%), and maleic anhydride (MA, 99%) were acquired from Merck. Wax and poly­(vinyl alcohol) (PVA) were both obtained from Chukyo Yushi Co. Ltd. No further purification was performed on any of the chemicals. All solutions were prepared using deionized (DI) water with a resistivity of 18.2 MΩ·cm.

### 2.2 Synthesis of the Additive

The synthesis protocol was adapted from our previously reported methodology for the preparation of PEG-grafted ceramic processing additives, with modifications to the monomer composition and reaction conditions for the present SiAlON system.^32–34^ The dough formation is attributed to polymer bridging between ceramic particles. On the other hand, the PEG-grafted architecture is expected to improve packing among the particles,^32,34^ resulting in a homogeneous coagulated structure with sufficient cohesion for handling and machining. The esterification process of PEG-1000 with MA was carried out through the procedure suggested by Lu et al.^40^ The synthesized MA-esterified PEG-1000 (MAPEG) was directly employed in the polymerization process without undergoing purification. For a typical aqueous free-radical copolymerization of AA/AMPS/MAPEG at a molar ratio of 1:1:0.3, 0.05 mol of AA, 0.05 mol of AMPS, and 0.015 mol of MAPEG (Mw ≈ 550 g·mol^–1^) were dissolved in 110 mL of deionized water. The solution pH was then adjusted to about 8 using an aqueous NaOH solution.

This mixture was then transferred to a 250 mL three-neck flask connected to a reflux condenser. Under nitrogen purge and magnetic stirring, the temperature was raised to 50 °C. Subsequently, a 10 mL solution of KPS (initiator, 1 mol % of the total moles of the monomers) was added dropwise to the reaction chamber over 50 min. Afterward, the temperature was increased to 60 °C, and a second 10 mL initiator solution was added. The temperature of the reaction was further raised to 80 °C, and the reaction continued for 4 h. After the reaction mixture was cooled down to room temperature, the copolymer was precipitated using ethanol and subsequently dried at 60 °C for 24 h. The copolymer composition (Figure S1, Table S1) was analyzed by nuclear magnetic resonance spectrometry (NMR, Varian Unity Inova 500 MHz, Agilent Technologies, USA). Molecular weight parameters (Mw, Mn) and polydispersity index were determined by gel permeation chromatography (GPC, Agilent 1260, USA) equipped with a refractive index detector and operated at a flow rate of 0.7 mL·min^–1^ (Table S2). For GPC analysis, the copolymer was dissolved in phosphate buffer (pH 7.2) at a concentration of 5 mg·mL^–1^.

### 2.3 Electrokinetic Studies

#### 2.3.1 Determination of Minimum Copolymer Amount

To determine the minimum copolymer amount, the electrokinetic characterization was focused solely on Si_3_N_4_ particles, which constitute the highest concentration (>90 wt %) within the multicomponent ceramic system. A zeta potential analyzer (Malvern Instruments, Ltd., Zetasizer Nano series, UK) equipped with a 633 nm laser and a scattered light detector set at a fixed angle of 173° was employed. To prepare dispersions, 1, 5, and 10 mg of particles were individually dispersed in 100 mL of deionized (DI) water, resulting in particle concentrations of 0.00001, 0.00005, and 0.0001 g·mL^–1^, respectively. Each dispersion underwent 5 min of ultrasonication in a bath sonicator to ensure thorough mixing and dispersion of the particles. Subsequently, an aqueous copolymer solution with a concentration of 0.0001 g·mL^–1^ was introduced into these suspensions to obtain copolymer concentrations ranging from 0.25 to 2.5 wt % relative to the amount of particles. The mixtures were sonicated for 5 min followed by 2 min of mechanical stirring. Three measurements were conducted at 25 °C, each comprising a minimum of 15 runs. The zeta potential and size at a specific copolymer concentration were determined as the averages of these three measurements.

#### 2.3.2 Determination of Particle Size

To determine the average size of the powder mixture that is to be shaped through die-pressing for the fabrication of the commercial part, Si_3_N_4_, CaCO_3_, Al_2_O_3_, Sm_2_O_3_, Y_2_O_3_, and AlN were milled and mixed in a slurry form using wax and PVA as pressing aids. The average particle size was measured via dynamic light scattering (DLS) as ∼0.8 μm in the sample that was taken from this slurry. In comparison, to determine the average particle size of the mixture that is used to prepare self-standing doughs, a suspension of the powder mixture was prepared at a concentration of 0.00001 g·mL^–1^ in DI water, not milled, and the average particle size was measured as ∼1.5 μm. Incidentally, the size of the as-received Si_3_N_4_ was found to be 230 nm through DLS.

### 2.4 Preparation of the Self-Standing, Malleable SiAlON Doughs

To identify the maximum solid loading that exhibits a dough-like consistency, we prepared a matrix with different particle loadings and additive contents (Figure 2d). An aqueous copolymer solution was added gradually to the preweighed ceramic particles, and the suspensions were hand-mixed for 2 min until a dough-like consistency was achieved (Video S1). Digital images of the suspensions were captured using a Canon EOS 650D camera (Figure S2). The suspension that had a 72.7 wt % particle loading and 2 wt % copolymer produced the most consistent results. A 100 g dough of this composition comprises 72.7 g of particles, 25.9 g of DI water, and 1.4 g of the copolymer. The volumetric composition of the dough was 46.39% (v/v) SiAlON, 2.73% (v/v) copolymer, and 50.88% (v/v) DI water. In all compositions, the copolymer amount is reported relative to the weight of the SiAlON particles.

**Figure 2:**
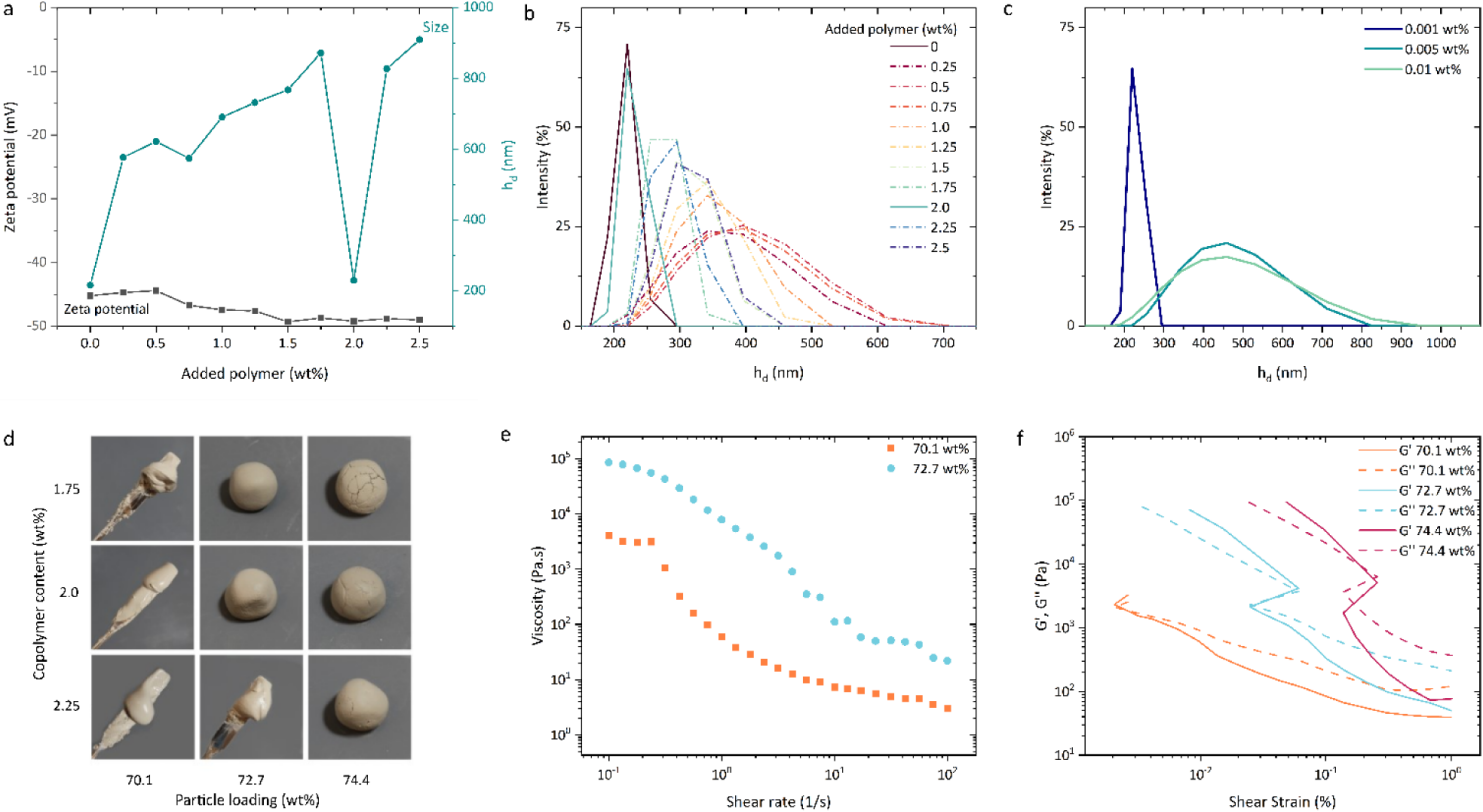
a) Zeta potential and *h*_d_ of the as-received Si_3_N_4_ particles (0.001 wt %), and in the presence of increasing copolymer content (line plot was used for enhanced data visualization); b) intensity-weighted size distribution of 0.001 wt % Si_3_N_4_ dispersion upon addition of the copolymer; c) intensity-weighted size distribution of 0.001–0.01 wt % Si_3_N_4_ dispersion upon addition of 2 wt % copolymer; d) the photography matrix displays the SiAlON suspensions at different compositions; e) viscosity as a function of shear rate; and f) oscillatory rheological response of SiAlON suspensions in the presence of 2 wt % of additive with varied solid loading (70.1, 72.7, and 74.4 wt %).

### 2.5 Preparation of the Green Bodies for the Fabrication of the Commercial-Grade Wear Part

A commercial-grade SiAlON composition with a molar ratio of 0.9Y-0.5Sm-0.5Ca was prepared by milling the composition in DI water with pressing aids.^36^ Wax and PVA were used as pressing aids, each at a concentration of 2.25 wt %, totaling 4.5 wt % relative to the weight of the powders. The milled slurry was spray-dried into granules.^36^ These granules were then used to fabricate die-pressed commercial-grade SiAlON wear parts with a pressing machine (Komage, S-XT). Incidentally, to avoid energy-intensive processes, we do not utilize milling for the preparation of self-standing doughs.

### 2.6 Rheological Characterization

An Anton Paar MCR92 rheometer equipped with a cone–plate geometry of 25 mm/2° (gap size: 0.103 mm) and a parallel plate geometry of 25 mm with a fixed gap size of 1 mm was used to characterize the rheological behavior of ceramic suspensions at room temperature (25 °C). Samples with 70.1, 72.7, and 74.4 wt % solid loading containing 2 wt % additive were prepared. The shear rate was varied from 0.1 to 100 s^–1^. To obtain the storage and loss moduli in dynamic mode, a parallel plate geometry (plate size 25 mm) was used at room temperature with a gap size of 1 mm. The angular frequency was kept constant at 10 rad·s^–1^, while the strain increased from 0.001 to 100%.

### 2.7 Green Machining of Self-Standing SiAlON Green Bodies

The green bodies were dried at room temperature (20.4 ± 1 °C) for 24 h under a relative humidity of 52%. Following the stabilization of the moisture content at ∼1.3% (Figure S3) after 18 h, subsequent machining operations were performed on the green body. The machining (Figure S4) was carried out using a benchtop 3-axis REVO M1A CNC milling machine (REVO, Turkiye), operating at a spindle speed of 10,000 rpm and a cutting speed of 1000 mm·min^–1^ (depth of cut: 0.2 mm). Solid carbide flat-end mill cutting tools with diameters of 1 mm and 2 mm (Kırtaş Sert Metal, Tuurkiye) were utilized for the machining process (Figure S5). The flat-end mill was selected to ensure a smooth surface finish for precision and dimensional accuracy.^41–44^ The material removal rate (mg·s^–1^) was then determined by tracking the weight loss during the machining process.

Optical microscopy (Sanqtid, China) and surface profilometry (Dektak 6M, USA) were employed to evaluate the quality of the machined surface before sintering. The surface roughness was determined by surface profilometry with a stylus-type profilometer equipped with a 12.5 μm tip radius, applying a force of 2 mg. Measurements were performed perpendicular to the machining direction over a travel distance of 7.5 mm (resolution: 0.833 μm/sample) for the machined surfaces.

### 2.8 Sintering Profile, Density Measurements, and Shrinkage

The green bodies were sintered by a two-step GPS cycle where the first step (presintering) was carried out at 1900 °C for 1 h at 5 bar nitrogen gas pressure, followed by a sintering step at 1950 °C for 1 h at 50 bar (FCT, FPW 180/2200, Germany). The density of the sintered bodies was determined using Archimedes’ method.

To assess the percentage of shrinkage during the sintering of green bodies, a K-factor was introduced to quantify the percentage of anisotropic sintering shrinkage using eqs 1–3:^45^

1 $$K_{xy}=100\times \left(1-\varepsilon_{x}/\varepsilon_{y}\right)$$

2 $$K_{xz}=100\times \left(1-\varepsilon_{x}/\varepsilon_{z}\right)$$

3 $$K_{yz}=100\times \left(1-\varepsilon_{y}/\varepsilon_{z}\right)$$

where ε_*x*_, ε_*y*_, and ε_z_ represent shrinkage in the *x*-, *y*-, and *z*- direction, respectively. The shrinkage in different directions was calculated as the average of nine cubical samples.

### 2.9 Microstructural Characterization

A comparison of microstructural properties was carried out with respect to the commercially available wear part that was manufactured by MDA Advanced Ceramics Inc. The phase ratios of the sintered samples were determined by X-ray diffraction spectroscopy (XRD, Rigaku Rint 2200, Japan) using Cu Kα radiation (λ = 1.540 Å) with a scanning speed of 1° 2θ/min between 20° and 40° 2θ angles. The phase ratios were found through a quantitative estimation from the XRD patterns using the integrated intensities of the (102) and (210) reflections of α-SiAlON and the (101) and (210) reflections of β-SiAlON using eq 4:

4 $$\frac{I_{\beta }}{I_{\beta }+I_{\alpha }}=\frac{1}{1+K\left[\left(\frac{1}{W_{\beta }}\right)-1\right]}$$

where *I*_α_ and *I*_β_ are the X-ray intensities of α- and β-SiAlON peaks, respectively, *W*_β_ is the relative weight fraction of β-SiAlON, and *K* is the constant resulting from eqs 5 and 6:

5 $$I_{\beta }=K_{\beta }\times W_{\beta }$$

6 $$I_{\alpha }=K_{\alpha }\times W_{\alpha }$$

which is 0.518 for β (101)−α (102) reflections and 0.544 for β (210)−α (210) reflections.^46^

Microstructural analysis of the sintered SiAlONs was performed via SEM (Zeiss-Supra 50 VP/Germany) on polished samples under backscattered electron imaging mode at an accelerating voltage of 20 keV with gold–palladium coating.

Tool wear was also assessed utilizing SEM (JEOL LSM 6010, Japan) at an accelerating voltage of 5–10 keV with no coating due to the conductive nature of the samples.

### 2.10 Mechanical Characterization

Vickers hardness (HV10) was evaluated using an indenter (Emco-Test Prüfmaschinen GmbH, Austria) with a 10 kg load applied for 5 s, following the ASTM-C1327 standard. Prior to testing, an automated polishing machine (TegraPol-25, Struers) was used to polish sintered (i) commercial-grade wear components and (ii) samples with a diameter of ∼16.2 mm and a thickness of ∼4.9 mm (shrunk from the original green body with a diameter of 20 mm and a thickness of 5 mm) that were prepared from a self-standing dough. Five indentations were made, and the fracture toughness of the samples was computed through the Niihara method.^47^

The flexural strength of both green (40 mm × 5 mm × 4 mm) and sintered (40 mm × 15 mm × 4 mm) SiAlON samples, produced from self-standing doughs, as well as a commercial-grade wear part (20 mm × 6 mm × 6 mm), was evaluated using a three-point bending configuration with a load cell capacity of 10 kN. For the samples produced from self-standing doughs, a span of 25 mm was used, while an 18 mm span was employed for the commercial-grade wear part. The tests were conducted using a Universal Testing Machine (Zwick/Roell Z010, USA). The testing speed was set to 0.2 mm·min^–1^ for the green samples and 1 mm·min^–1^ for the sintered samples.

## 3 Results and Discussion

### 3.1 Formulating Machinable SiAlON Green Bodies

Zeta potentiometry was used to evaluate the stability of very dilute Si_3_N_4_ suspensions in the presence of different amounts of the copolymer to determine the minimum amount of additive to prepare a dough (Figure 2a). The initial Si_3_N_4_ suspension (0.001 wt %), in its pristine form (without the additive), displayed a zeta potential (ζ) of −45 mV at pH ∼7. With the introduction of the additive, the zeta potential first increased to −44 mV up to 0.5 wt % then decreased eventually reaching a plateau at ∼−49 mV, indicating complete particle coverage^48^ at 2 wt %. Concurrently, the hydrodynamic diameter (*h*_d_) initially decreased, followed by an increase beyond 2 wt % additive. Ceramic particles had the smallest *h*_d_ (230 nm) in the presence of 2 wt % copolymer, aligning with the zeta potentiometry for the full surface coverage of particles. The upward trend in *h*_d_ (Figure 2a and b) with polymer dosages exceeding 2 wt % is attributed to mechanisms such as polymer–polymer/free-polymer interactions and bridging flocculation.^49–53^ Moreover, a wider size distribution and an *h*_d_ of ∼600 nm at higher solid loadings (0.005 and 0.01 wt %) were observed with 2 wt % copolymer (Figure 2c, Table S3).

We also prepared a matrix that is composed of suspensions with 70.1, 72.7, and 74.4 wt % particle loading and 1.75, 2, and 2.25 wt % additive (Figure 2d). At 70.1 wt %, all suspensions flowed rather than formed self-standing doughs. Although 74.4 wt % loading produced doughs at 1.75 and 2 wt % additive content, the doughs exhibited cracks. Overall, we singled out 72.7 wt % solid loading and 2 wt % additive content to formulate SiAlON doughs.

### 3.2 The Flow Behavior of SiAlON Suspensions/Doughs

Homogeneous coagulation without any bleeding is essential for the preparation of the dough. We characterized the flow behavior of the ceramic suspensions (70.1, 72.7, and 74.4 wt %) in the presence of 2 wt % additive. While tracking steady-state shear viscosity, the 74.4 wt % suspension crumbled upon applying the shear force and spread out of the measuring plate, hence assessing its flow behavior was not possible. The structural breakdown, especially with higher solid loading, is attributed to drying during the measurement. 70.1 wt % suspension showed a paste-like behavior (Figure 2d and e) with a relatively lower viscosity compared to the 74.4 wt % counterpart. At shear rates ranging from 0.1 to 100 s^–1^, both 70.1 and 72.7 wt % SiAlON suspensions displayed shear-thinning behavior. For the 70.1 wt % suspension, the viscosity remained below 50 Pa·s in the measurement range and the system formed an “ink” rather than a dough—this formulation can be extruded from a syringe with ease. In contrast, the 72.7 wt % SiAlON-loaded formulation exhibited a dough-like behavior across shear rates of 0.1 to 100 s^–1^. For instance, there is a ∼10-fold difference in viscosity at the shear rate of 10 s^–1^ between 72.7 and 70.1 wt % loading, and sustaining a viscosity over 100 Pa·s is critical to achieve a malleable dough. Figure 2f displays the viscoelastic properties of suspensions with different solid loadings. All suspensions displayed elastic behavior where the storage modulus is higher than the loss modulus (*G*′ > *G*″). The yield stress and viscosity of these two formulations were consistent with the particle loading.

### 3.3 Green Machining of SiAlONs

The SiAlON dough had a flexural strength of 0.5 MPa (Figure S7), demonstrating its ability to withstand machining forces and maintain its structural integrity. To assess the performance of self-standing doughs during machining, tools with two different diameters, 1 and 2 mm, were used. The material removal rate for SiAlON with a 1 mm tool was estimated as ∼6.46 mg·s^–1^, while employing a 2 mm tool yielded a rate of ∼12.32 mg·s^–1^. Figure 3a,b,e, and f show the optical images of the green compacts after machining. When machining a SiAlON surface at its green state using a 2 mm cutting tool (Figure 3f), a higher degree of damage is observed at the edges compared to the case of a 1 mm cutting tool (Figure 3b). The machinability of the material was evaluated by tracking wear at the cutting edge of the tool as well. In Figure 3d and h, SEM images of the clearance faces of the tools following the machining of the SiAlON compacts are presented; no tool wear was observed in either case. The optical images of the tools used in machining are also provided in Figure S5.

**Figure 3:**
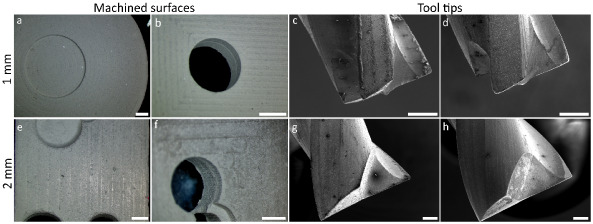
a) SiAlON disc; b) close-up of a hole machined using a 1 mm flat-end tool; SEM micrograph of the 1 mm tool c) before use and d) after use; e) SiAlON surface; f) close-up of a hole machined using a 2 mm flat-end tool; SEM micrograph of the 2 mm tool g) before use, and h) after use. Scale bars represent 2 mm for optical images (a, b, e, and f) and 250 μm for SEM micrographs.

The averages of arithmetic surface roughness were 85 ± 4.6 and 76 ± 5.5 μm for the surfaces machined with 1 and 2 mm diameter tools, respectively (Figure S6). The larger diameter of the 2 mm tool offers a broader contact area during machining, resulting in more uniform material removal and a smoother finish, hence a lower surface roughness.^54,55^ Overall, employing a larger diameter tool for machining achieved a smoother surface, and a small diameter tool performed better at drilling with lower edge damage.

Importantly, the low flexural strength of the self-standing dough facilitated successful machining without observable tool wear or significant edge damage.

### 3.4 Microstructural and Mechanical Characterization

Following GPS, molded objects had a linear shrinkage of ∼19% from the dried state. The average *K*_*xy*_, *K*_*xz*_, and *K*_*yz*_ were calculated as −1.04, −2.45, and 1.40 using eqs 1–3, respectively. A large absolute value of the K-factor implies higher shrinkage anisotropy, and *K* ≥ 12 was associated with anisotropic shrinkage; thus, a K-factor of ∼2 in our system is considered isotropic.^45^ The postsintering density was ∼3.24 g·cm^–3^, reaching ∼99.99% of theoretical density.

Figure 4a and b show SEM backscattered electron images of the sintered commercial-grade wear part and the one prepared from the dough, respectively. In Figure 4a and b, dark grains represent β-SiAlON, gray grains specify α-SiAlON while white spots indicate the intergranular phase due to the sintering additives. The larger grains in SiAlON (Figure 4b) that are prepared from self-standing doughs are attributed to the absence of milling of the powders before the preparation of the dough. For the commercial-grade product, XRD patterns (Figure 4c) have distinct peaks corresponding to both α-SiAlON and β-SiAlON phases, consistent with the observed dark and gray grains in Figure 4a. XRD analysis of the self-standing SiAlON, which predominantly consists of β-SiAlON, shows sharp diffraction peaks corresponding primarily to the β phase. The destabilization of the small amount of α phase, the reduction in the crystallization degree, and the alteration in the chemical composition of the grain boundary phase suggest an increase in oxygen content within the system. This phenomenon is likely caused by the hydrolysis of AlN.^56^

**Figure 4:**
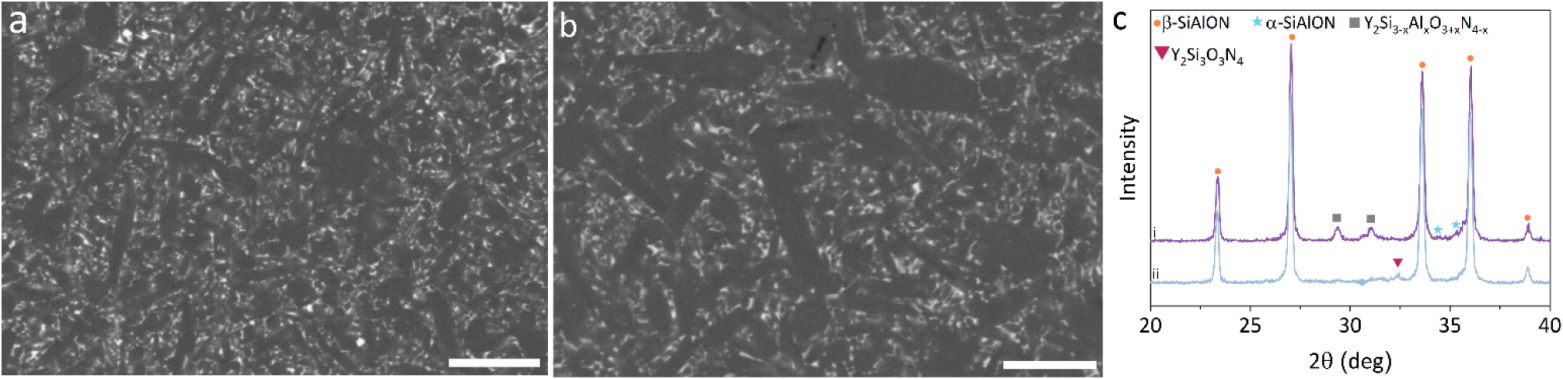
SEM backscattered electron micrographs of a) a commercial-grade wear part; b) SiAlON part prepared from the dough (Scale bars represent 10 μm); and c) XRD spectra of (i) commercial-grade and (ii) self-standing SiAlON.

The flexural strength of the sintered self-standing SiAlON was calculated to be ∼243 MPa, with a Weibull modulus of 4.52, indicating moderate variability in its strength (Figure S8a and b), whereas the commercial-grade SiAlON exhibited a higher flexural strength of ∼319 MPa (Figure S8b). We calculated the hardness and extrapolated indentation fracture toughness of sintered self-standing SiAlONs as 13.48 ± 0.35 GPa and 6.22 ± 0.28 MPa·m^1/2^, respectively. These values are in alignment with the commercial-grade reference values of 14.40 ± 0.39 GPa and 6.40 ± 0.40 MPa·m^1/2^. We believe the finer particle size of the commercial-grade wear part contributed to its higher mechanical strength and hardness.

In addition, the hardness (13.48 ± 0.35 GPa) and fracture toughness (6.22 ± 0.28 MPa·m^1/2^) obtained in this study compare constructively with values reported for conventionally processed SiAlON ceramics (hardness ≈ 14 GPa, toughness ≈ 5 MPa·m^1/2^) and additively manufactured SiAlONs (hardness ≈ 16.7 GPa, toughness ≈ 4.84 MPa·m^1/2^).^8,57^

### 3.5 Fabrication of Custom-Based Shapes

The fabrication of complex shapes that include holes, threads, and grooves is presented to highlight the capability of the process. A gear, a four-tooth comb, a clover, and a spline were prototyped through CNC machining (Figure 5). Overall, the process enables laboratories with limited materials engineering expertise to engage in ceramic processing for prototyping and low-volume fabrication without requiring expensive setups, aligning with the goals of affordability and accessibility.

**Figure 5:**
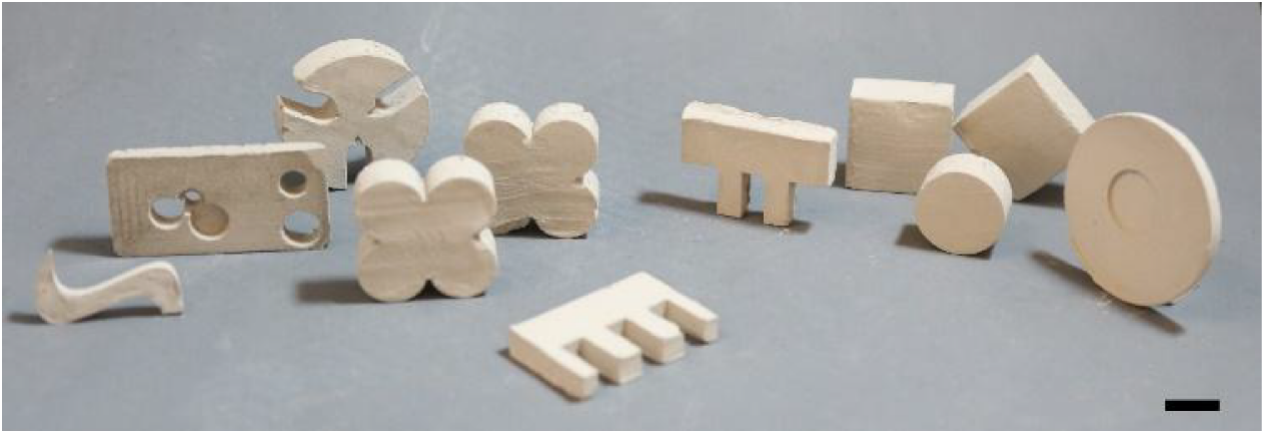
Custom-sized shapes at their green state after machining (Scale bar represents 1 cm).

## 4 Conclusion

Manufacturing advanced ceramics in complex geometries remains a challenge due to their inherent brittleness. Although large-scale production benefits from established methods, these routes can be inadequate for rapid prototyping, where the cost of molds and equipment imposes significant limitations. The proposed approach, which employs dough-like ceramic formulations to prepare a green body, provides a viable alternative to conventional routes, overcoming existing constraints and enabling more straightforward pathways for low-volume fabrication.

Despite exhibiting significantly lower mechanical strength compared with other green body formulations, these doughs are malleable and sustain their structural integrity during conventional machining; therefore, they require lower machining forces and impose only insignificant tool wear. Here, the preparation of the first malleable dough of a multicomponent nitride ceramic system and the demonstration of the feasibility of machining at the green state are reported. Importantly, this processing flexibility is achieved without sacrificing the final performance, as the sintered bodies reached near-theoretical density and exhibited mechanical properties comparable to those of conventionally processed SiAlON ceramics. The expansion from oxides to nitrides and to a multicomponent system underlies the ease-of-use and possible universality of the approach with clear implications for lowering the barriers for scalable fabrication of advanced materials.

## Supplementary Material





## Data Availability

Data will be made available on request.

## Notes

^1^H NMR spectrum of the copolymer, preparation of the SiAlON green bodies, moisture content and machining of the SiAlON green bodies, tool wear, average surface roughness, flexural strength of SiAlON bodies (PDF)
Process flow of the fabrication of complex-shaped objects from malleable, self-standing doughs (MP4)
