# Supplementary material for: Self-Standing Doughs of SiAlONs Enable Low-Volume Production through Green Machining
Source: ACS Omega. 2026 Jul 23;11(30):45528–35. doi: 10.1021/acsomega.6c05186 (PMC13448937; doi:10.1021/acsomega.6c05186)
Supplement: Supplementary file 1 [file ao6c05186_si_001.pdf]

SUPPORTING INFORMATION

Self-standing doughs of SiAlONs enable low-volume production through green machining

Ayse Ay[a], Gokhan Kula[b], Burcin Gul[a], Ceylin Isiklar[a], Eminenur Saritas[a], Alpagut Kara[b,c], and Ozge Akbulut[a]*

[a]Faculty of Engineering and Natural Sciences, Sabanci University, Istanbul, 34956, Turkiye

[b]MDA Advanced Ceramics Inc., 75.Yil OSB Mah., Anadolu Cad., No: 2, Odunpazari, Eskisehir 26250, Turkiye

[c]Department of Materials Science and Engineering, Eskisehir Technical University, Eskisehir, 26555, Turkiye

***Corresponding author at: Sabancı University, Orta Mah., Universite Cad., No: 27, Tuzla, Istanbul, Turkiye. ozge.akbulut@sabanciuniv.edu**

Calculation of monomer ratios by Nuclear magnetic resonance spectroscopy (NMR)

The peaks used for calculating the molar ratio of additives are identified as follows: i) 3.6-3.8 ppm (corresponding to CH_2-CH_2-O of PEG side chains, with 92 protons), ii) 3.4-3.6 ppm (representing S-CH_2 of AMPS monomer, with 2 protons), and iii) 1.9-2.3 ppm (-CH of AA + -CH of AMPS + -CH-CH of MAPEG of side chain, denoting AA monomer). The molar feed ratio was computed as follows: i) PEG (CH_2-CH2-O) with an integral value of 2.62 and associated value of 0.028, ii) AMPS (S-CH_2) with an integral value of 20.42 and associated value of 10.21, and iii) AA+AMPS+PEG (-CH) with an integral value of 44.58, resulting in an associated value of 44.58-10.21-0.056=33.71 for AA. Thus, the side chain density (PEG/AA+AMPS) is determined as 0.028/44.52=0.0006, and AMPS/AA is calculated as 10.21/34.71=0.3. The peaks between 4.39–3.98 ppm represent possible impurities of free PEG, bi-functional and mono-functional PEG, and a hydroxyl dimer of PEG diol [1].

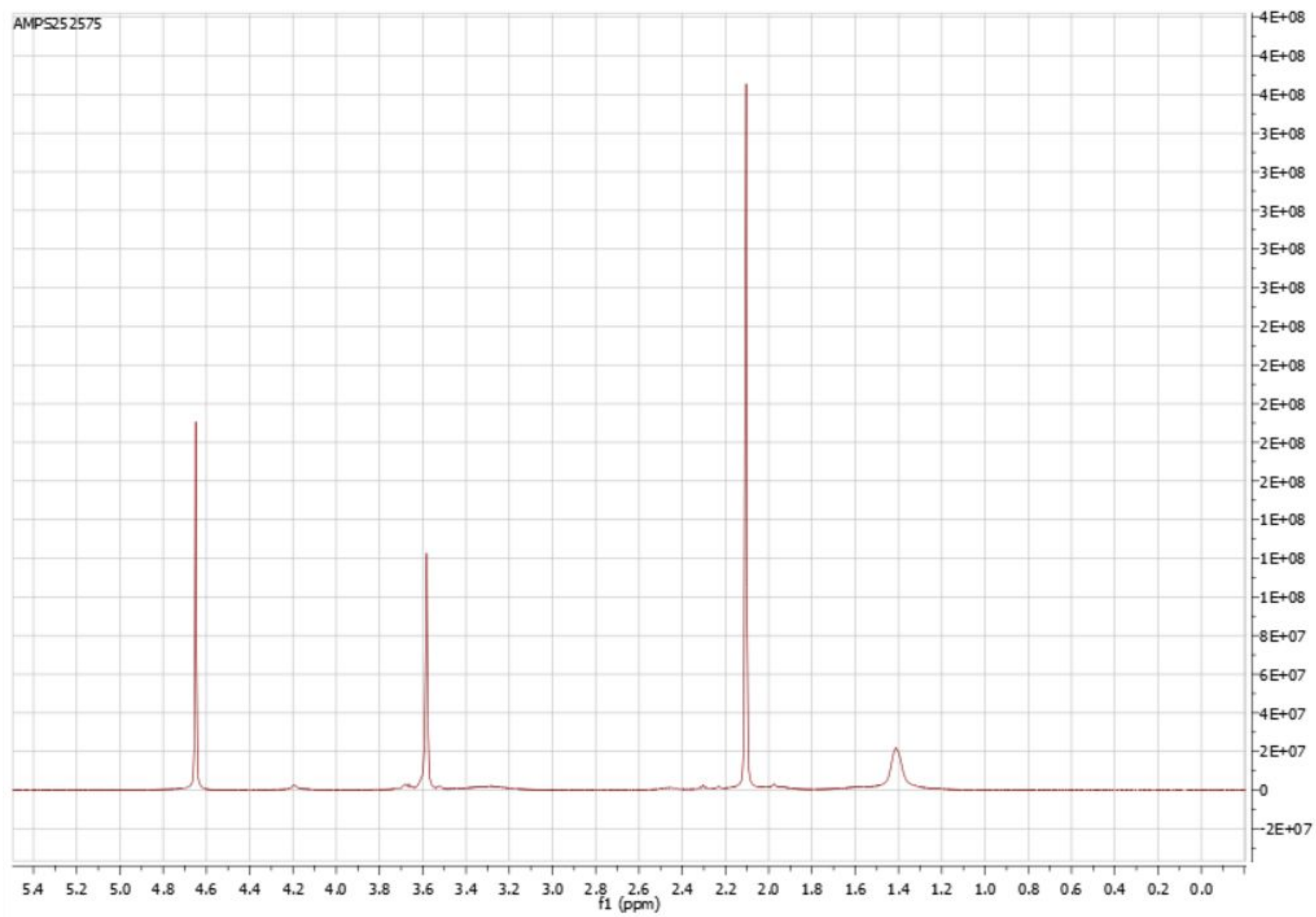

Figure S1. ^{1}H NMR spectrum of the AMPS/AA/PEG copolymer in deuterium oxide (D_2O) (The peak at ~4.65 ppm originates from D_2O solvent in the measurement.)

Table S1. ^{1}H NMR absolute integral values associated with each functional group of the copolymer

Region	Range	Functional group	Absolute Integral
1	4.39–3.98		2.17
2	3.84–3.63	CH_2-CH_2-O	2.60
3	3.62–3.48	S-CH_2	20.42
4	3.47–3.12		5.63
5	2.80–2.58		0.48
6	2.34–1.81	-CH, -CH-CH	44.58
7	1.80–1.50		4.26
8	1.51–1.36		18.17
9	1.33–1.25		1.69

Table S2. Properties and characteristics of the copolymer

Molar concentration (AMPS:AA:PEG)	0.05:0.05:0.015
Molar feed ratio (AMPS:AA:PEG)[a]	1:1:0.3
AMPS/AA[a]	3:10
PEG/AMPS+AA[a]	0.06:100
Molecular weight (M_n) (kg·mol^{-1})[b]	111.285
PDI (M_w/M_n)[b]	2.11

[a] Determined by ^{1}H-NMR, [b] Determined by gel permeation chromatography (GPC)

Coagulation study - assessing the particle-polymer interaction

Polydispersity indices (PDI) of size measurements are provided in Table S3.

Table S3. Copolymer concentration with respect to Si_3N_4 particles (0.001 wt%) and their corresponding PDIs of intensity-weighed size distribution

Copolymer concentration (wt%)	PDI
0	0.863
0.25	0.456
0.5	0.540
0.75	0.504
1.0	0.557
1.25	0.597
1.5	0.620
1.75	0.737
2.0	0.825
2.25	0.699
2.5	0.728

Preparation of the green bodies

Figure S2 shows the preparation of a malleable, self-standing dough comprising 72.7 wt% SiAlON and 2 wt% copolymer.

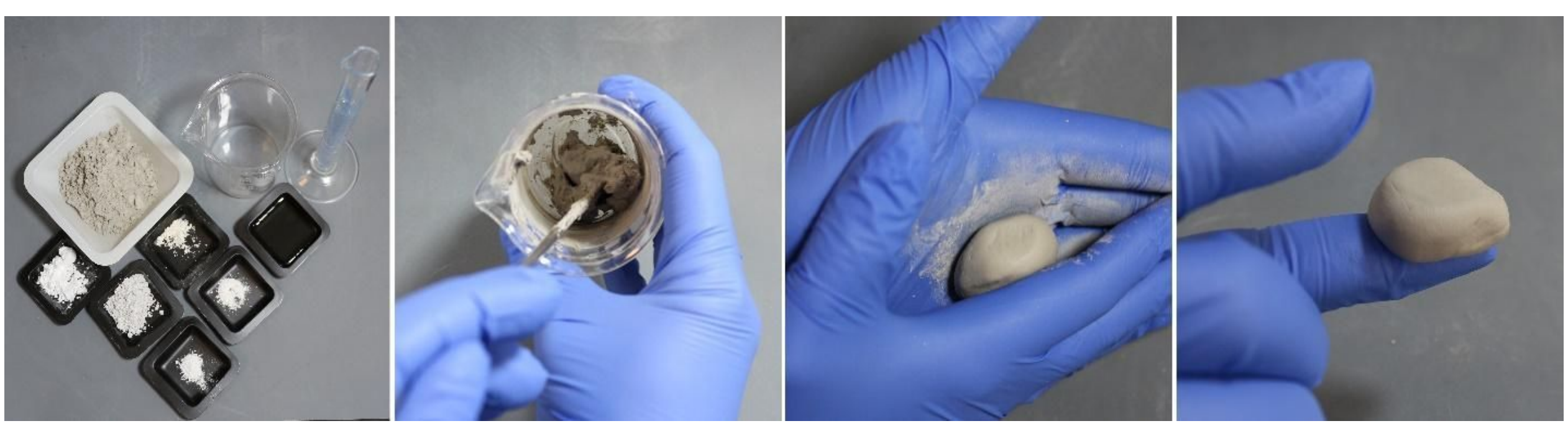

Figure S2. The preparation steps of a malleable, self-standing dough comprising 72.7 wt% SiAlON and 2 wt% copolymer

Tracking the moisture content of green bodies

All hand-shaped objects demonstrated linear shrinkage of ~2.2 % from molding to the dried state. To assess machinability, we monitored the moisture content of the molded doughs over a 24-hour period of drying (Figure S3) under ambient conditions (temperature: 20.4 ± 1 °C, relative humidity: 52 %) using a solid content analyzer.

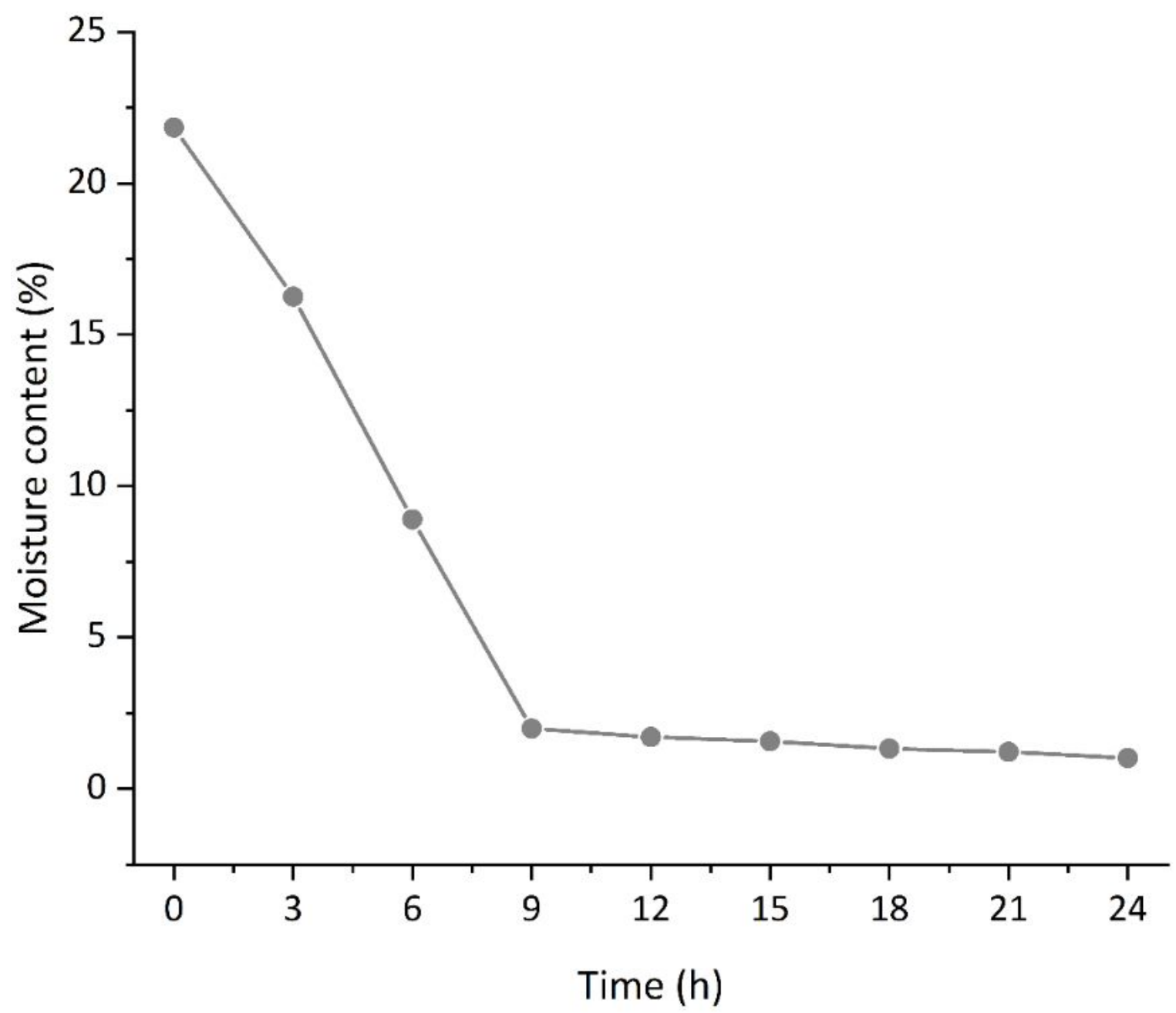

Figure S3. The moisture content of the dough that contains 72.7 wt% SiAlON and 2 wt% copolymer (with respect to SiAlON) as a function of time during drying under ambient conditions

Green machining of SiAlONs

The setup equipped with a 2 mm flat end mill ready to machine a cylinder is provided in Figure S4. The green body is fixed to the machining table using adhesive tape.

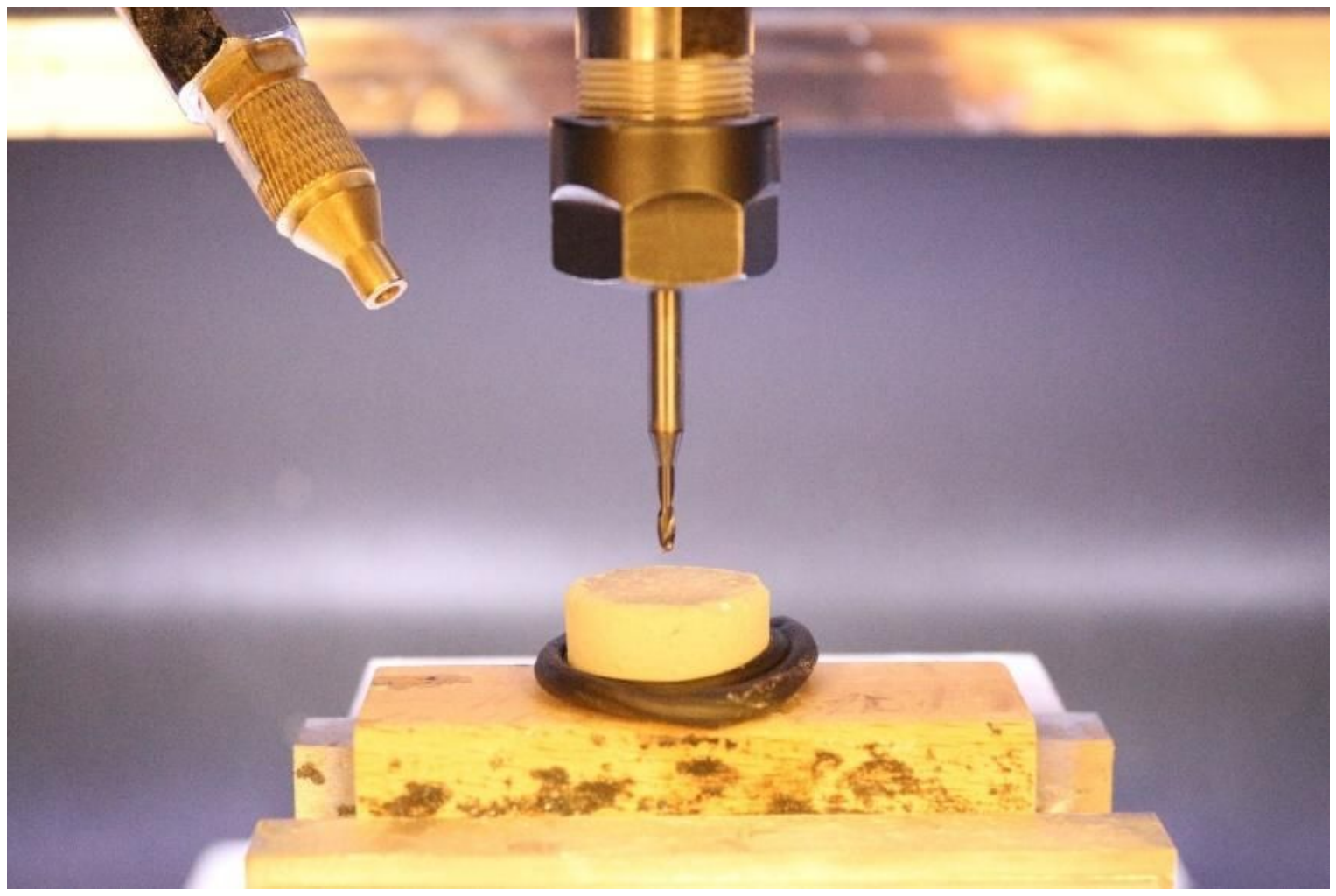

Figure S4. The setup for mounting the sample onto the sample holder for CNC machining

The tool wear associated with 4 hours of use is provided in Figure S5 and no significant wear was observed for both tools.

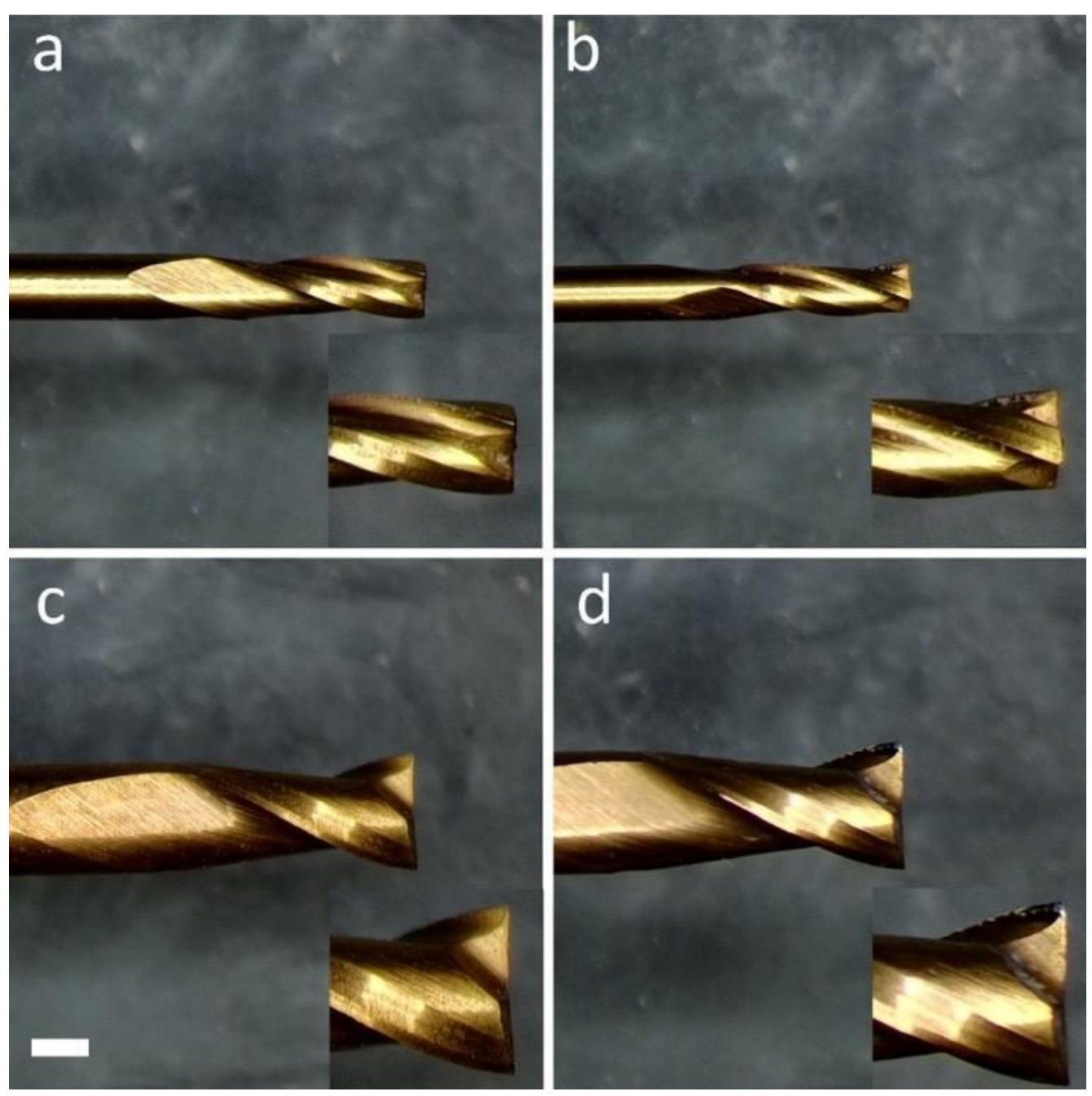

Figure S5. a) A new 1 mm diameter cutting tool before use, b) its wear after 4 hours of use, c) a 2 mm diameter cutting tool, d) its wear after 4 hours of use (The images in the lower right corners provide magnified views of the cutting tools, with zoom factors of a) 1.6x, b) 2.1x, c) 1.5x, and d) 1.5x. The scale bar in every image represents 1 mm.)

Figure S6 presents the surface roughness of machined surfaces using different tools, evaluated through profilometry.

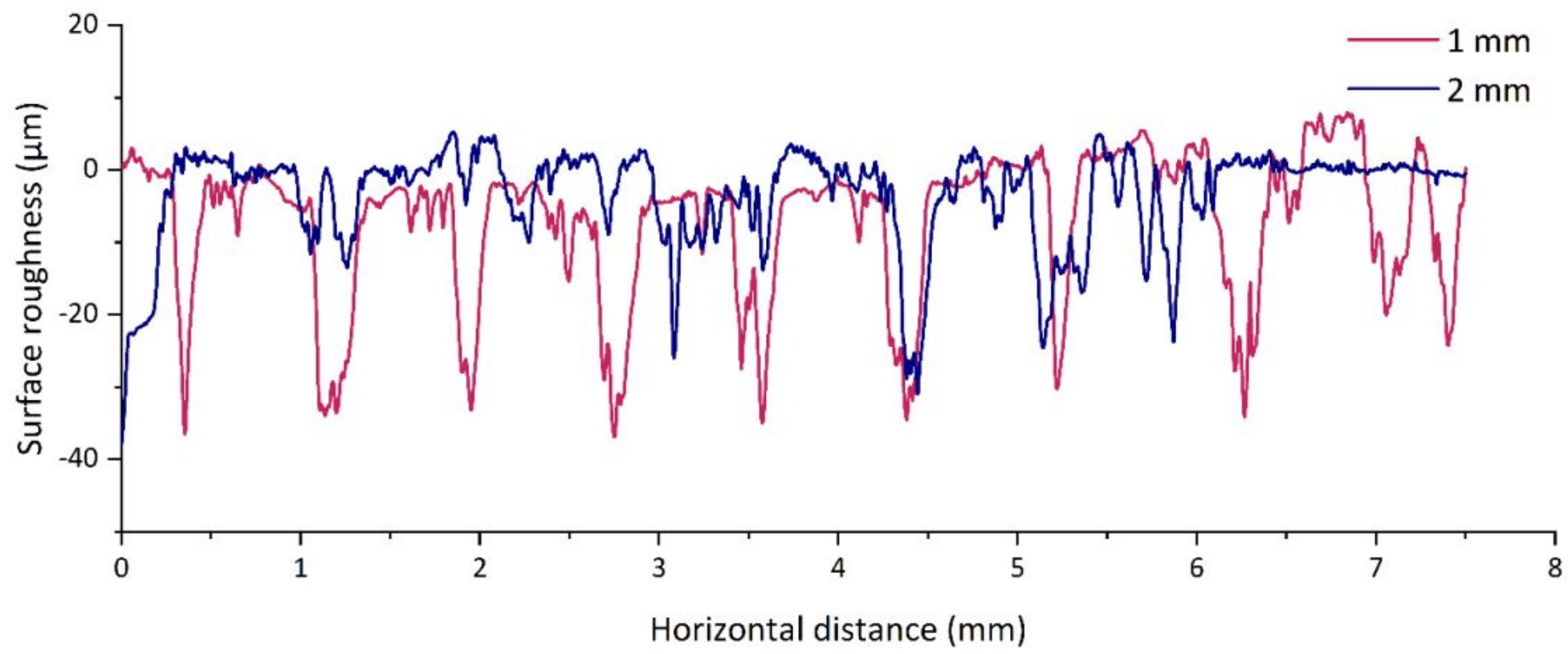

Figure S6. Surface roughness of the SiAlON surfaces that are machined with 1 and 2 mm tools

Flexural strength of SiAlON green bodies

The flexural strength of green SiAlON samples (40 mm × 5 mm × 4 mm) was assessed using a three-point bending configuration with a 25 mm span, employing a Universal Testing Machine (Zwick/Roell Z010, USA) at a testing speed of 0.2 mm·min^{-1}. The flexural modulus and flexural strength are estimated as ~1.5 MPa and ~0.5 MPa, respectively (Figure S7).

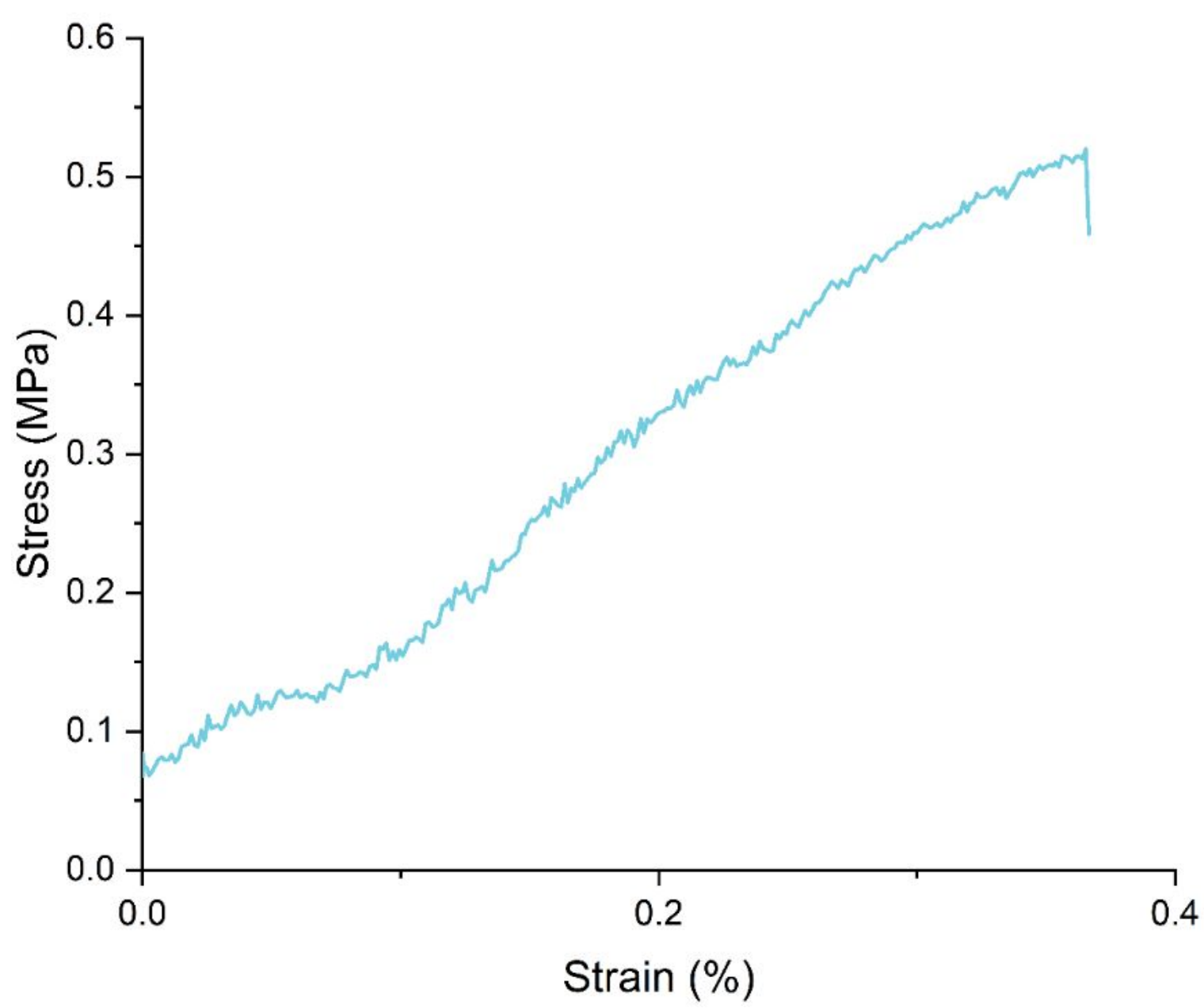

Figure S7. Stress-strain curve of green SiAlON

The flexural strength of sintered (40 mm × 15 mm × 4 mm) SiAlON samples, produced from self-standing doughs and a commercial-grade wear part (20 mm × 6 mm × 6 mm), was evaluated using a three-point bending configuration with a load cell capacity of 10 kN (Figure S8). For the samples produced from self-standing doughs, a span of 25 mm was used, while an 18 mm span was employed for the commercial-grade wear part. The tests were conducted using a Universal Testing Machine (Zwick/Roell Z010, USA). The testing speed was set to 1 mm·min^{-1}.

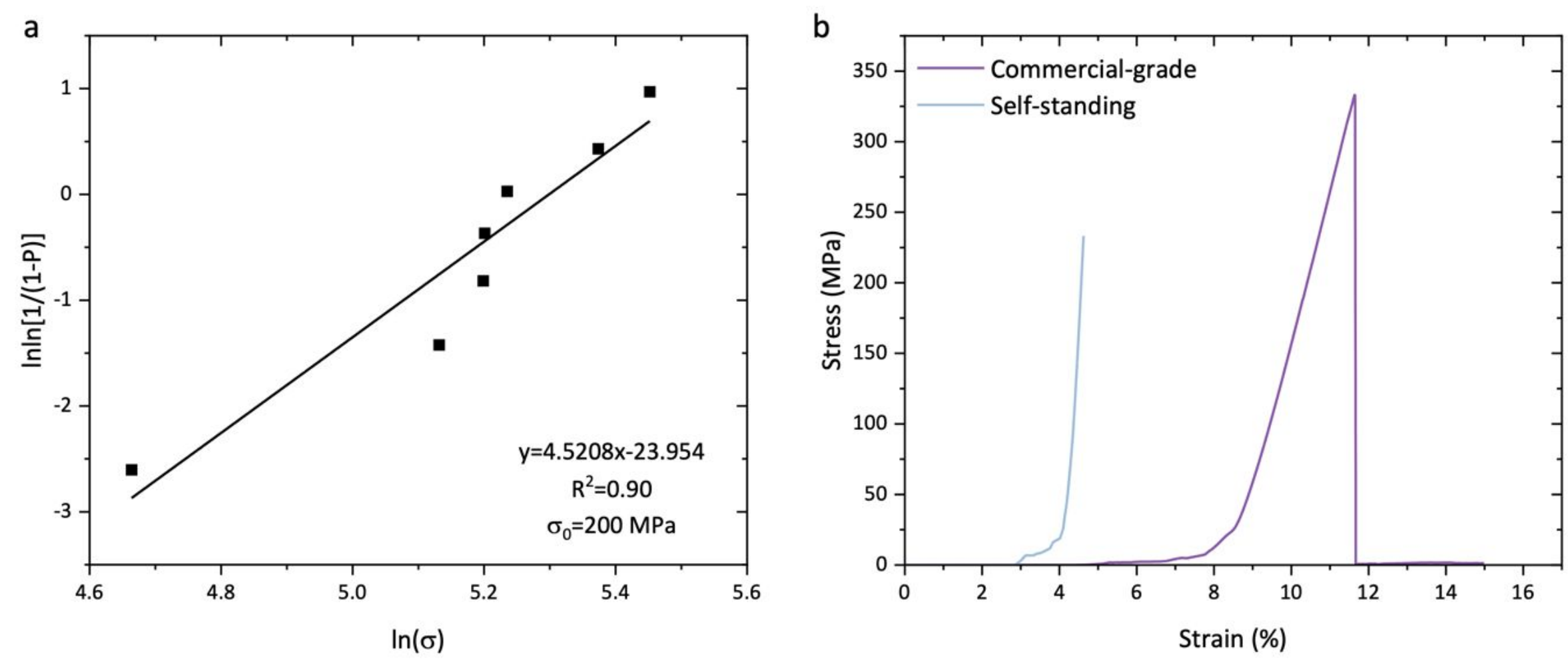

Figure S8. a) Weibull distribution analysis of the flexural strength of sintered SiAlON produced from the self-standing dough formulation and b) stress-strain curves comparing the sintered state of the samples that are produced via pressing (commercial-grade) versus those produced from self-standing doughs

References

1 Harrell M. L., and Bergbreiter D. E., Using 1H NMR Spectra of Polymers and Polymer Products To Illustrate Concepts in Organic Chemistry, *Journal of Chemical Education*, **2017**, 94, 1668–1673 DOI: 10.1021/acs.jchemed.6b00801
